# Microglial-Targeted GCPII Inhibition Reverses Neurocognitive Impairment and Synaptic Loss After EcoHIV Infection

**DOI:** 10.3390/cells15060502

**Published:** 2026-03-12

**Authors:** Yuxin Zheng, Meixiang Huang, R. Michael Maragakis, Peter Pietri, Yu Su, Jesse Alt, Lukáš Tenora, Wathsala Liyanage, Ying Wu, Mary-Anne Thomas, Rangaramanujam M. Kannan, Xiaolei Zhu, Rana Rais, Barbara S. Slusher

**Affiliations:** 1 Department of Infectious Diseases, The Second Xiangya Hospital of Central South University, Changsha 410011, China; yzhen124@jh.edu; 2 Johns Hopkins Drug Discovery, Johns Hopkins University School of Medicine, Baltimore, MD 21205, USA; mhuang52@jhmi.edu (M.H.); rmaraga1@jhmi.edu (R.M.M.); ppietri1@jh.edu (P.P.); ysu46@jh.edu (Y.S.); jalt1@jhmi.edu (J.A.); ltenora1@jhu.edu (L.T.); ywu58@jhmi.edu (Y.W.); mthom231@jh.edu (M.-A.T.); xzhu31@jhmi.edu (X.Z.); rrais2@jhmi.edu (R.R.); 3 Department of Neurology, Johns Hopkins University School of Medicine, Baltimore, MD 21205, USA; 4 Center for Nanomedicine, Department of Ophthalmology, Wilmer Eye Institute, Johns Hopkins University School of Medicine, Baltimore, MD 21231, USA; wghmliy1@jhmi.edu (W.L.); krangar1@jhmi.edu (R.M.K.); 5 Department of Chemical and Biomolecular Engineering, Johns Hopkins University, Baltimore, MD 21218, USA; 6 Hugo W. Moser Research Institute, Kennedy Krieger, Inc., Baltimore, MD 21205, USA; 7 Kennedy Krieger Institute–Johns Hopkins University for Cerebral Palsy Research Excellence, Baltimore, MD 21218, USA; 8 Department of Psychiatry and Behavioral Sciences, Johns Hopkins University School of Medicine, Baltimore, MD 21205, USA; 9 Department of Oncology, Johns Hopkins University School of Medicine, Baltimore, MD 21205, USA; 10 Department of Physiology, Pharmacology and Therapeutics, Johns Hopkins University School of Medicine, Baltimore, MD 21205, USA; 11 Department of Medicine, Johns Hopkins University School of Medicine, Baltimore, MD 21205, USA; 12 Department of Neuroscience, Johns Hopkins University School of Medicine, Baltimore, MD 21205, USA

**Keywords:** dendrimer, GCPII, HIV, EcoHIV, NAAG, glutamate, cognitive impairment, synaptic loss

## Abstract

**Highlights:**

**What are the main findings?**

- D-2-PMPA preferentially accumulates in brain microglia and increases NAAG levels in EcoHIV-infected mice.
- D-2-PMPA reverses EcoHIV-induced cognitive, social, and synaptic deficits.

**What is the implication of the main findings?**

- Targeting microglial GCPII represents a promising approach for treating HIV-associated neurocognitive disorders.

**Abstract:**

HIV-associated neurocognitive impairment persists despite combination antiretroviral therapy, largely driven by chronic microglial activation that sustains neuroinflammation and neuronal injury. Activated microglia contribute to HIV-associated brain pathology by releasing proinflammatory mediators that disrupt synaptic integrity and impair cognition. N-acetylaspartylglutamate (NAAG), an abundant neuropeptide that maintains glutamatergic homeostasis, is hydrolyzed by glutamate carboxypeptidase II (GCPII) to glutamate. We previously demonstrated that reduced brain and cerebrospinal fluid NAAG levels in people living with HIV correlate with cognitive impairment, and that pharmacological GCPII inhibition with 2-(phosphonomethyl)-pentanedioic acid (2-PMPA) elevates brain NAAG and improves cognition in EcoHIV-infected mice. To enhance brain delivery and preferentially target activated microglia, we conjugated 2-PMPA to a generation 4 hydroxyl poly(amidoamine) (PAMAM) dendrimer (D-2-PMPA). Our findings demonstrate that D-2-PMPA achieves preferential microglial drug delivery, resulting in a >600% increase in cerebrospinal fluid NAAG levels. At doses 8.3-fold lower than free 2-PMPA, this formulation reversed EcoHIV-induced deficits in social interaction, novel object recognition, and fear-conditioned memory without altering locomotor activity or anxiety-like behavior. D-2-PMPA also restored prefrontal cortex synaptic density and preserved dendritic architecture. Together, these findings demonstrate that microglia-targeted GCPII inhibition represents a potent nanotherapeutic strategy to restore synaptic integrity and cognitive function in HIV-associated neurocognitive impairment.

## 1. Introduction

Human Immunodeficiency Virus (HIV), a member of the lentivirus family, targets the human immune system. Over time, HIV infection leads to acquired immunodeficiency syndrome (AIDS), wherein weakened immunity predisposes individuals to potentially fatal opportunistic infections. Although combination antiretroviral therapy (cART) markedly improves survival and viral suppression, people living with HIV (PLH) continue to experience neurocognitive deficits, most commonly affecting working memory and executive function, underscoring an unmet need for mechanism-based neurotherapeutic strategies [1,2,3,4].

The neuropeptide N-acetyl-aspartyl-glutamate (NAAG) plays a key role in regulating glutamatergic synaptic activity and neuroinflammation. NAAG is one of the most abundant neuropeptides in the brain and signals primarily through activation of metabotropic glutamate receptor 3 (mGluR3), leading to suppression of glutamate release and attenuation of excitotoxic and inflammatory signaling [5,6]. The enzyme glutamate carboxypeptidase (GCPII) metabolizes the neuropeptide NAAG into N-acetylaspartate (NAA) and glutamate [7]. Previous clinical studies have demonstrated a significant correlation between brain NAAG levels and cognition in PLH, with higher NAAG levels associated with better cognitive function, suggesting that impaired NAAG signaling may contribute to cognitive dysfunction [8,9].

Microglia, the resident innate immune cells of the central nervous system (CNS), are long-lived, self-renewing, and uniquely susceptible to HIV-1 infection [10,11,12,13,14,15]. These properties position microglia as persistent viral reservoirs capable of sustaining chronic neuroinflammation even under cART [16]. Post-mortem and transcriptomic analyses in PLH have revealed that brain macrophages and microglia are the cells harboring HIV DNA in the brain [17,18], and cognitive impairments were associated with greater microgliosis [19]. In addition, recent findings from human cerebral organoid models indicate that HIV-infected microglia exhibit elevated secretion of pro-inflammatory cytokines and chemokines, thereby fostering an inflammatory and neurotoxic microenvironment that leads to neuronal dysfunction and subsequent cell death [20,21]. Together, these findings implicate microglial dysregulation as a central driver of neuroHIV pathology.

We previously showed that pharmacological inhibition of GCPII using 2-(phosphonomethyl)-pentanedioic acid (2-PMPA) increased brain NAAG and significantly improved cognitive performance in EcoHIV-infected mice [22]. However, the clinical translation of GCPII inhibition is limited by the poor brain penetration and lack of cell specificity of small-molecule inhibitors. Notably, GCPII activity is measurably upregulated in microglia following neuroinflammation [23], demonstrating that microglia are a key site for GCPII-mediated regulation in the CNS. Given the central role of microglia in neuroHIV, we hypothesized that targeted delivery of a GCPII inhibitor to microglia would enhance therapeutic efficacy.

To test this hypothesis, we conjugated 2-PMPA to generation-4 hydroxyl-terminated poly(amidoamine) (G4-PAMAM-OH) dendrimers (D-2-PMPA) [23]. Hydroxyl-dendrimers have been shown by us and others to preferentially deliver small molecule therapeutics, including GCPII inhibitors, to microglia [23,24,25,26,27]. Herein we evaluated the microglial targeting, ex vivo GCPII inhibition, and therapeutic efficacy of D-2-PMPA in EcoHIV-infected mice, with the goal of establishing a microglia-directed strategy to increase brain NAAG and ameliorate HIV-associated neurocognitive dysfunction.

## 2. Materials and Methods

### 2.1. Synthesis of D-2-PMPA and Cy5-Labeled Dendrimer–Conjugated 2-PMPA (Cy5-D-2-PMPA)

The detailed synthesis and full characterization of D-2-PMPA and Cy5-D-2-PMPA are reported elsewhere [23,28]; a concise summary is provided here. D-2-PMPA was synthesized via partial esterification of a PAMAM-OH dendrimer with hexynoic acid using EDC/DMAP coupling to introduce approximately 11 surface alkyne functionalities, followed by rigorous purification by dialysis. The alkyne-functionalized dendrimer was then conjugated to 2-PMPA-PEG11-azide through CuAAC click chemistry to afford D-2-PMPA. Successful conjugation was conclusively confirmed by 1H NMR and HPLC analyses, which indicated an average of ~11 2-PMPA molecules per dendrimer (corresponding to ~10 wt% drug loading) and a final purity ~99.5%. D-2-PMPA exhibited a hydrodynamic diameter of 4.7 nm and a near-neutral surface charge (ζ-potential −2.07 mV). Using an analogous synthetic strategy, a fluorescently labeled Cy5-D-2-PMPA conjugate was also prepared [23,28].

### 2.2. Animals

Male C57BL/6J mice were purchased from Jackson Laboratory (Bar Harbor, ME, USA), and housed in a temperature- and humidity-controlled environment on a 14-h light/10-h dark cycle with ad libitum access to food and water. Only male mice were used to minimize variability associated with estrous cycle-dependent behavioral and immunological differences. All experimental protocols involving animals received prior approval from the Johns Hopkins University Animal Care and Use Committee and adhered to established institutional guidelines. Mice were randomly assigned to experimental groups, and all behavioral, histological, and biochemical analyses were performed by investigators blinded to treatment conditions.

### 2.3. EcoHIV Infection

EcoHIV was prepared as previously described [29,30]. Briefly, HEK293T cells were transfected with the EcoHIV/NDK plasmid, kindly provided by Dr. Volsky’s lab at Icahn School of Medicine at Mount Sinai. Viral particles were harvested from culture supernatant via centrifugation, and viral concentrations were quantified using a p24 ELISA kit (Advanced Bioscience Laboratories, 5421, Rockville, MD, USA). Ten-week-old male C57BL/6 mice were randomly divided into three experimental groups (*n* = 10 per group). Two groups were inoculated intraperitoneally (i.p.) with EcoHIV virus (4 × 10^6^ pg per mouse), whereas the control group received an equivalent volume of 1× phosphate-buffered saline (PBS, ThermoFisher, 10010023, Waltham, MA, USA). Successful infection was confirmed by evaluating p24 antigen levels in splenic homogenates using a p24 ELISA kit at 3 days post-inoculation using a separate cohort of mice (*n* = 3).

### 2.4. Dosing Paradigm

#### 2.4.1. Co-Localization Studies

To evaluate in vivo dendrimer distribution, a separate cohort of EcoHIV-infected mice (*n* = 5) was used. Three weeks post-infection, mice received a single i.p. injection of Cy5-D-2-PMPA (20 mg/kg, 2-PMPA equivalent) and were euthanized 24 h after injection.

#### 2.4.2. Efficacy Studies

Three weeks post-infection (13 weeks of age), mice were randomized to treatment groups and dosing was initiated on an every-other-day schedule. Treatment group animals were administered D-2-PMPA (20 mg/kg, expressed as 2-PMPA equivalent) via i.p. injection at 10 mL/kg, formulated in HEPES-buffered saline (Corning, 25-060-CI, Corning, NY, USA) and administered at a volume of 10 mL/kg. D-2-PMPA was synthesized as previously described [23]. Control animals received HEPES-buffered saline at matching volumes and dosing intervals. The same vehicle control group was used in our prior study of 2-PMPA in EcoHIV-infected mice [22]. Treatments continued for two weeks, during which body weight was monitored weekly.

### 2.5. Behavioral Studies

Before each behavioral test, mice were acclimatized for 30 min in their home cage in the behavioral testing room. All behavioral arenas were cleaned thoroughly with Vimoba disinfectant between each mouse to minimize odor cues. A series of behavioral evaluations was conducted 24 h post-final treatment, comprising the Open Field Test (OFT), Light–Dark Box (LDB), Novel Object Recognition Test (NORT), Social Interaction Test (SIT), and Fear Conditioning Test (FCT), administered sequentially [31,32,33,34,35].

#### 2.5.1. OFT

Locomotor activity was assessed using a San Diego Instruments Photobeam Activity System–Open Field (PAS-Open Field). The arena (41 cm × 41 cm × 38 cm, W × D × H) consisted of four transparent acrylic walls and a gray stainless-steel floor. A 16 × 16 photobeam frame with 1-inch beam spacing surrounded the enclosure. Locomotor activity was recorded as total beam breaks over 30 min.

#### 2.5.2. LDB

Anxiety-like behavior was assessed using a light–dark box apparatus. The apparatus comprised a square enclosure (40 cm × 40 cm × 35 cm, W × D × H) divided into 2 equal chambers by a black partition with a small opening (7 cm × 7 cm). One chamber (light side) was made of transparent plastic and was brightly illuminated (100–200 lux), whereas the other chamber (dark side) was made of black infrared-permeable perspex and dimly lit (4–7 lux). Mice were placed in the light chamber at the start of the test and allowed to freely explore both chambers. Time spent in each chamber over 10 min was recorded and analyzed.

#### 2.5.3. NORT

Recognition memory was evaluated over two days, consisting of three phases: habituation, training, and testing. On the first day (habituation phase), mice were placed individually into the testing chamber without objects and allowed to freely explore for 30 min. 24 h later, mice were placed into the same apparatus (training phase) containing two identical objects (matched for color, size, material, and shape) placed along the diagonal of the chamber and allowed to explore for 10 min. After a 30-min interval, the test phase was conducted: one of the familiar objects was replaced with a novel object that differed only in shape, and mice freely explored both objects for 5 min. An overhead video camera recorded animal behavior, and blinded observers quantified object exploration duration. The recognition index (RI) represented the proportion of novel object exploration time relative to total exploration time, while the discrimination index (DI) was calculated as the difference between novel and familiar object exploration times divided by total exploration time.

#### 2.5.4. SIT

Sociability and social novelty were assessed using a three-chamber apparatus (40 cm × 26 cm × 20 cm, W × D × H), containing empty wire cups in the side chambers, over four consecutive 10-min trials: habituation, exploration, sociability, and social novelty preference. During the habituation phase, mice were individually placed in the middle chamber and were blocked from accessing either of the side chambers. In the exploration phase, mice were allowed to access all three chambers. During the sociability trial, an age-, sex-, and strain-matched unfamiliar mouse was placed in one wire cup, while an inanimate object was placed in the other. In the social novelty preference trial, the inanimate object was replaced by a second unfamiliar mouse, and mice were allowed to freely explore all chambers. The amount of time spent in each chamber and around each cup (sniffing) was recorded and quantified using ANY-maze tracking software (v7.49; Stoelting Co., Wood Dale, IL, USA).

#### 2.5.5. FCT

Associative fear memory was evaluated over three consecutive days using a Med Associates NIR Video Fear Conditioning (VFC) system. On the first day (training phase), mice were placed in the chamber and allowed to explore freely for 150 s. They were then subjected to two tone-shock pairings: a conditioned stimulus (CS) tone (2000 Hz, 80 dB, 30 s) immediately followed by an unconditioned stimulus (US) foot shock (0.5 mA, 2 s), with a 30-s interval between pairings. Twenty-four hours after training, contextual fear memory was tested for 5 min in the same chamber. On the third day, cued fear memory was tested with an altered context. Three US-only stimuli were delivered to mice at 120, 180, and 240 s during a 5 min testing session. Freezing responses were recorded throughout all experimental phases.

### 2.6. NAAG Analysis in Cerebrospinal Fluid (CSF)

CSF samples were collected from the cisterna magna immediately after euthanasia following 2 weeks of treatment and completion of behavioral testing and stored at −80 °C until analysis. Each group initially included 10 mice, and CSF samples were successfully collected from 9 mice per group. Quantification of NAAG concentrations was performed using liquid chromatography coupled with tandem mass spectrometry (LC-MS/MS). This method was specifically optimized for analysis of low-volume CSF samples, as previously reported [22]. Briefly, Calibration standards were prepared by spiking NAAG into artificial CSF (0.119 M NaCl, 0.026 M NaHCO_3_, 2.5 mM KCl, 1 mM NaH_2_PO_4_, 1.3 mM MgCl_2_, 10 mM glucose) to generate a standard curve spanning 0.01–100 µM in half-log increments. Five microliters of each CSF sample or standard were transferred to low-retention microcentrifuge tubes. Protein precipitation was performed by adding 200 µL of methanol containing 250 nM deuterated NAAG (NAAG-d_3_) as an internal standard, followed by vortex mixing and centrifugation at 16,000× *g* for 5 min at 4 °C. The resulting supernatants were used for LC-MS/MS analysis. Chromatographic separation was achieved using an UltiMate 3000 ultra-high-performance liquid chromatography system coupled to a Q Exactive Focus Orbitrap mass spectrometer (ThermoFisher Scientific). An amide-based hydrophilic interaction liquid chromatography (HILIC) column (Waters Acquity UPLC BEH Amide, 1.7 µm, 2.1 × 150 mm; Waters Corporation, Milford, MA, USA) was used for analyte separation. The mobile phases consisted of water supplemented with 0.1% formic acid (A) and acetonitrile containing 0.1% formic acid (B), delivered using a gradient elution. NAAG quantification was performed in positive-ion mode using product-ion monitoring with a collision energy of 10 CE. The monitored transitions for NAAG were *m*/*z* 305.1 → 130.0498, 148.0603, and 158.0449, while NAAG-d_3_ was monitored at *m*/*z* 308.1 → 133.1168 and 161.0638. Data acquisition and quantitative analysis were conducted using Xcalibur 4.7 SP1 software (ThermoFisher Scientific, Waltham, MA, USA).

### 2.7. Immunofluorescence Staining

Mice were euthanized using CO_2_ and then transcardially perfused first with PBS, followed by 4% paraformaldehyde (PFA). Brains were collected and processed for cryosectioning as previously described [36,37]. Following overnight post-fixation in 4% PFA at 4 °C, brains were cryoprotected in sequential sucrose solutions (15% and 30%), embedded in O.C.T. compound, and snap-frozen. Coronal cryosections (20 μm) were prepared for immunofluorescence staining. Sections were PBS-rinsed, permeabilized, and blocked in TBS with 4% goat serum, 2% BSA, and 0.2% Triton X-100 for 1 h at room temperature. Overnight incubation at 4 °C followed with primary antibodies in blocking buffer: anti-MAP2 (rabbit, 1:500; Millipore, AB5622-I, Burlington, MA, USA; dendrites), anti-synaptophysin (mouse, 1:500; CST, 9020S, Danvers, MA, USA; presynaptic vesicles), anti-Iba1 (rabbit, 1:500; CST, 17198S, Danvers, MA, USA; microglia), anti-GFAP (chicken, 1:500; Abcam, ab4674, Cambridge, UK; astrocytes), and anti-NeuN (rabbit, 1:500; Abcam, ab177487, Cambridge, UK; neurons). Secondary antibodies included goat anti-rabbit Alexa Fluor 488 (1:1000; Invitrogen, A11034, Waltham, MA, USA) for MAP2, Iba1, and NeuN; goat anti-mouse Alexa Fluor 647 (1:1000; Invitrogen, A21240, Waltham, MA, USA) for synaptophysin; and goat anti-chicken Alexa Fluor 488 (1:1000; Invitrogen, A11039, Waltham, MA, USA) for GFAP. Sections were mounted with ProLong Glass Antifade Mountant with NucBlue stain (ThermoFisher, P36985, Waltham, MA, USA). Confocal z-stack images were acquired using an Andor BC43 microscope (Oxford Instruments, Abingdon, UK). For image processing and visualization, z-stacks were imported into Imaris (v10.2.0, Oxford Instruments, Abingdon, UK), and identical display settings (minimum/maximum intensity scaling) were applied across all images within each channel. Maximum-intensity projections were generated for each channel, and regions of interest (ROIs) were drawn. Quantification of fluorescence intensity within ROIs was performed in ImageJ (v2.16.0/Fiji, National Institutes of Health, Bethesda, MD, USA) using a constant threshold across all images; background signal (measured from an adjacent ROI lacking specific staining) was subtracted from each measurement.

### 2.8. Statistical Analysis

GraphPad Prism 10.6.0 (San Diego, CA, USA) was utilized for all statistical analyses. CSF NAAG level comparisons were performed using unpaired Student’s *t*-tests, whereas two-way ANOVA followed by Tukey’s multiple comparisons test was employed for all remaining outcome measures. Unless otherwise specified, data are presented as mean ± SEM.

## 3. Results

### 3.1. D-2-PMPA Is Preferentially Taken up by Microglia in EcoHIV-Infected Mice

**Figure 1:**
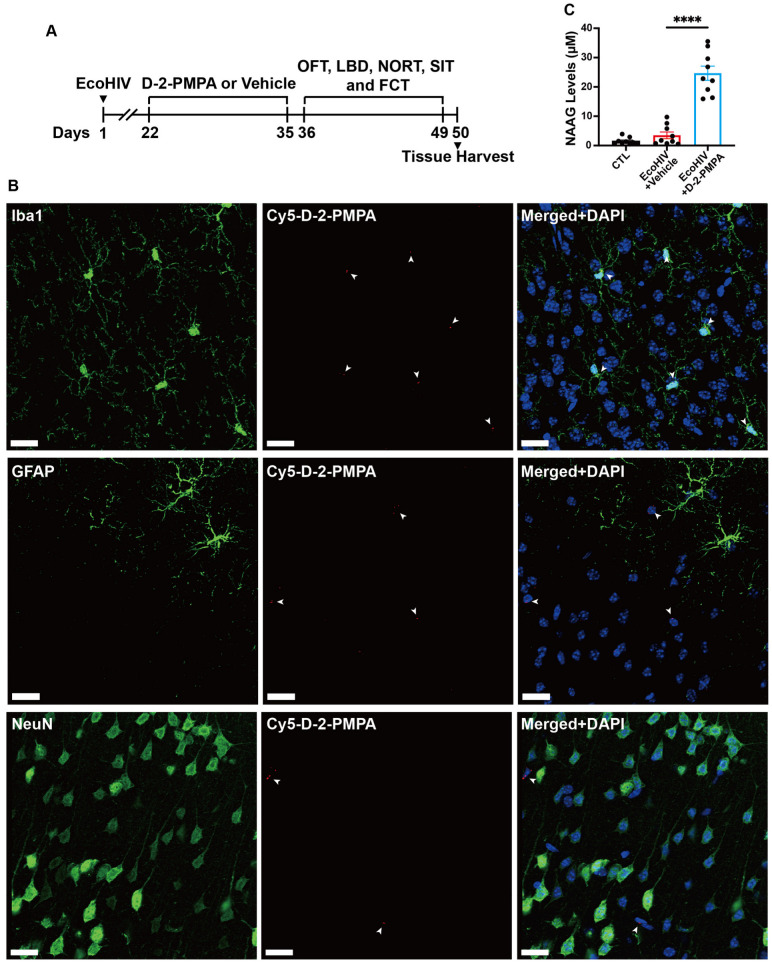
(**A**) Schematic of the study design. (**B**) Representative cortex images showing Cy5-D-2-PMPA (red, arrows) is co-localized with Iba1-positive microglia (green), but not co-localized with GFAP-positive astrocytes (green) or NeuN-positive neurons (green). Nuclei were counterstained with DAPI (blue). Scale bars = 20 μm (*n* = 5). (**C**) CSF NAAG levels were significantly elevated by D-2-PMPA treatment in EcoHIV-infected mice. (*n* = 9 per group). **** *p* < 0.0001.

To investigate the cellular distribution of dendrimer-conjugated GCPII inhibitor in the brain, Cy5-D-2-PMPA was administered via i.p. injection to 8-week-old EcoHIV-infected mice at three weeks post-infection. Twenty-four hours after administration (20 mg/kg, 2-PMPA equivalent), mice were euthanized and brain sections were immunostained for Iba1 (microglia), GFAP (astrocytes), and NeuN (neurons). Confocal imaging demonstrated that Cy5-D-2-PMPA signals predominantly colocalized with Iba1-positive microglia, with limited signal detected in GFAP- or NeuN-positive cells (Figure 1B).

### 3.2. D-2-PMPA Increases Brain NAAG Levels >600% in EcoHIV-Infected Mice

The effect of dendrimer-mediated GCPII inhibition on in vivo NAAG levels was evaluated in EcoHIV-infected mice treated with vehicle or D-2-PMPA (20 mg/kg, 2-PMPA equivalent, i.p.) every other day for two weeks, starting three weeks post-infection (Figure 1A). LC-MS/MS analysis of CSF revealed that D-2-PMPA administration significantly increased NAAG levels in the CSF (from 3.49 ± 1.13 µM to 24.67 ± 2.41 µM), corresponding to an approximately 607% increase (*p* < 0.0001; Figure 1C). This finding is consistent with our previously reported effect of GCPII inhibition [22].

### 3.3. D-2-PMPA Improves Recognition and Learning Memory Without Affecting Motor Function

**Figure 2:**
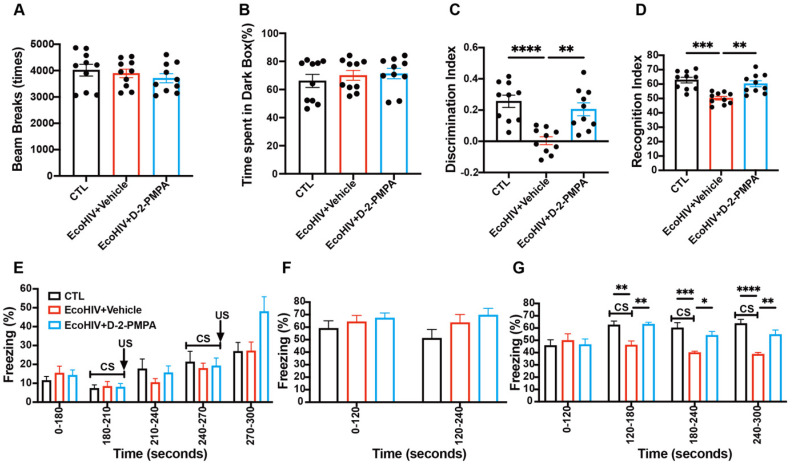
D-2-PMPA reverses cognitive and cued fear memory impairments without altering locomotor activity or anxiety-like behavior in EcoHIV-infected mice. (**A**) Total beam breaks during the open field test (OFT) were not significantly different among experimental groups. (**B**) Time spent in the dark compartment of the light–dark box (LDB) test remained comparable across all groups. (**C**,**D**) Novel object recognition test (NORT) analysis revealed that EcoHIV-infected mice exhibited significant deficits in both discrimination index (DI = (novel object time—familiar object time)/total exploration time) and recognition index (RI = novel object time/total exploration time) compared with uninfected controls (CTL); D-2-PMPA treatment effectively restored both measures. (**E**) Day 1 training phase in the fear conditioning test (FCT; conditioned stimulus (CS) tone: 180–210 s, 240–270 s; unconditioned stimulus (US) foot shock: 210 s, 270 s) showed no group differences in freezing. (**F**) Day 2 contextual fear memory in the FCT was similar across groups. (**G**) Cued fear memory, evaluated on Day 3 during the CS presentation period, was significantly attenuated in EcoHIV-infected mice; this deficit was effectively reversed by D-2-PMPA treatment. * *p* < 0.05, ** *p* < 0.01, *** *p* < 0.001, **** *p* < 0.0001, *n* = 10/group.

Eight-week-old mice were administered vehicle or EcoHIV, and after three weeks of infection, EcoHIV-infected mice were treated with either D-2-PMPA (20 mg/kg, 2-PMPA equivalent) or HEPES for two weeks before behavioral testing. OFT and LDB assessments demonstrated no significant group differences in locomotor activity (total beam breaks, Figure 2A) or anxiety-like behavior (time in dark compartment, Figure 2B). NORT analysis revealed that EcoHIV infection significantly impaired object recognition memory, as evidenced by reduced DI (*p* < 0.0001, Figure 2C) and RI (*p* < 0.001, Figure 2D) compared to uninfected controls. These cognitive deficits were significantly attenuated by D-2-PMPA treatment (DI: *p* < 0.01, Figure 2C; RI: *p* < 0.01, Figure 2D). In addition, fear-associated learning and memory were assessed using the FCT. On day 1, all groups displayed comparable freezing responses during tone–shock pairing, indicating equivalent acquisition of the conditioned response (Figure 2E). On day 2, contextual fear memory did not differ significantly between EcoHIV-infected mice and uninfected controls (Figure 2F). In contrast, during the cue-dependent fear memory test on day 3 during CS presentation, EcoHIV-infected mice exhibited a significant impairment in freezing response when compared to the uninfected control group. (120–180 s: *p* < 0.01; 180–240 s: *p* < 0.001; 240–300 s: *p* < 0.0001; Figure 2G), whereas freezing behavior was significantly increased in D-2-PMPA–treated EcoHIV-infected mice (120–180 s: *p* < 0.01; 180–240 s: *p* < 0.05; 240–300 s: *p* < 0.01; Figure 2G).

### 3.4. D-2-PMPA Rescues Social Behavior Deficits in EcoHIV-Infected Mice

**Figure 3:**
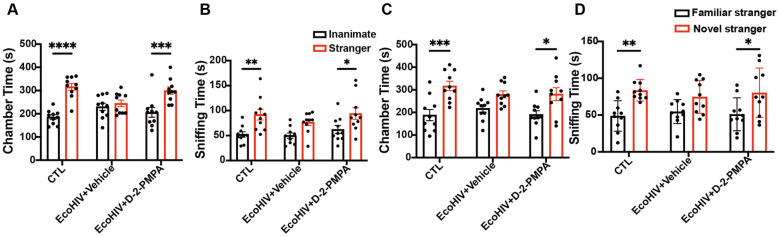
Sociability and social novelty deficits in EcoHIV-infected mice are reversed by D-2-PMPA treatment. Chamber time (**A**,**C**) and sniffing time (**B**,**D**) were evaluated. (**A**,**B**) The sociability test indicated that EcoHIV infection reduced social interaction behavior compared with uninfected controls; D-2-PMPA normalized these deficits. (**C**,**D**) Social novelty preference assessment revealed impaired recognition of novel social stimuli in EcoHIV-infected mice, which was rescued following D-2-PMPA administration. * *p* < 0.05, ** *p* < 0.01, *** *p* < 0.001, **** *p* < 0.0001, *n* = 10/group.

Next, sociability and social novelty preference were assessed using the three-chamber SIT. During the sociability phase, uninfected control mice spent significantly more time interacting with a stranger mouse than with an inanimate object, as measured by both chamber occupancy (*p* < 0.0001; Figure 3A) and sniffing duration (*p* < 0.01; Figure 3B). In contrast, EcoHIV-infected mice failed to show a preference for the stranger mouse over the inanimate object (Figure 3A,B), indicating impaired sociability. Treatment with D-2-PMPA restored this preference, with treated EcoHIV-infected mice spending significantly more time with the stranger mouse than with the inanimate object (chamber time: *p* < 0.001; sniffing time: *p* < 0.05; Figure 3A,B). During the social novelty preference test, uninfected control mice preferentially interacted with a novel stranger mouse compared with a familiar one, as reflected by increased chamber time (*p* < 0.001; Figure 3C) and sniffing time (*p* < 0.01; Figure 3D). EcoHIV-infected mice did not discriminate between the novel and familiar stranger mice (Figure 3C,D), whereas D-2-PMPA treatment EcoHIV-infected mice showed significantly greater interaction with the novel stranger mouse (chamber time: *p* < 0.05; sniffing time: *p* < 0.05; Figure 3C,D).

### 3.5. D-2-PMPA Reverses Synaptic Deficits in EcoHIV-Infected Mice

**Figure 4:**
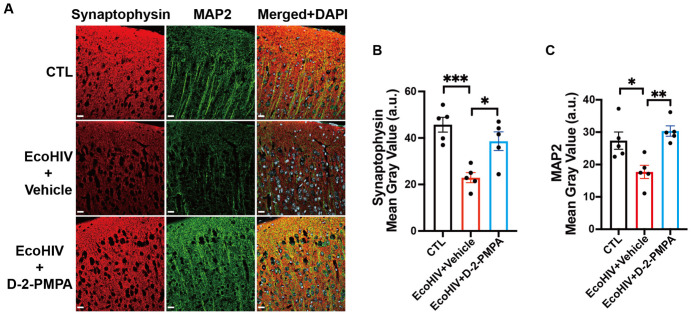
D-2-PMPA rescued synaptic and dendritic deficits in the cortex of EcoHIV-infected mice. (**A**) Representative cortical immunofluorescent images of synaptophysin (red) and microtubule-associated protein 2 (MAP2; green). Nuclei were counterstained with DAPI (blue). Scale bars = 20 μm. (**B**) Synaptophysin intensity quantification revealed synaptic dysfunction in EcoHIV-infected mice, reversed by D-2-PMPA. (**C**) Quantification of cortical MAP2 intensity showed reduced dendritic integrity in EcoHIV-infected mice, which was restored following D-2-PMPA administration. * *p* < 0.05, ** *p* < 0.01, *** *p* < 0.001, *n* = 5/group.

Following completion of behavioral testing, mice were euthanized, and neuronal integrity and synaptic structure were assessed by immunostaining brain sections for the dendritic marker MAP2 and the presynaptic vesicle protein synaptophysin (Figure 4A). Quantitative analysis of immunoreactivity revealed a significant reduction in cortical synaptophysin and MAP2 staining in EcoHIV-infected mice compared with uninfected controls (synaptophysin: *p* < 0.001, Figure 4B; MAP2: *p* < 0.05, Figure 4C), indicating synaptic and dendritic loss. Treatment with D-2-PMPA significantly restored both synaptophysin and MAP2 levels in EcoHIV-infected mice (synaptophysin: *p* < 0.05, Figure 4B; MAP2: *p* < 0.01, Figure 4C), consistent with preservation of synaptic integrity.

## 4. Discussion

The advent of cART has dramatically extended life expectancy and reduced CNS opportunistic infections in HIV-infected individuals; nevertheless, neurocognitive impairment remains a significant and prevalent burden among PLH [38]. Recent studies indicate that approximately 42–52% of virally suppressed PLH (VS-PLH) continue to exhibit cognitive impairment, with higher prevalence in individuals with greater comorbidity burden [39,40,41,42]. These persistent cognitive deficits adversely affect quality of life, daily functioning, and long-term outcomes, highlighting the need for adjunctive therapeutic strategies that target CNS dysfunction in VS-PLH.

A growing body of evidence supports a strong association between NAAG signaling and cognitive function across neurological diseases [43,44]. Clinical studies using in vivo magnetic resonance spectroscopy and post-mortem analyses demonstrated that reduced brain NAAG levels correlate with disease severity and/or cognitive performance in Alzheimer’s disease, multiple sclerosis, schizophrenia, and Huntington’s disease [45,46,47,48,49,50]. Consistent with these observations, preclinical studies show that GCPII inhibition raises brain NAAG levels and improves cognitive performance in animal models of multiple sclerosis, Alzheimer’s disease, traumatic brain injury, schizophrenia, and ethanol intoxication [23,49,51,52,53]. GCPII inhibition has also demonstrated neuroprotective effects in ischemic injury models and has been shown to promote remyelination in peripheral nerve injury [54,55,56]. Relevant to this study, recent clinical studies report that higher brain or CSF NAAG levels are associated with improved neurocognitive performance in PLH [8,9].

In the context of HIV, our prior in vitro studies showed that GCPII inhibition increases NAAG levels and mitigates gp120-induced neurotoxicity [57]. Consistent with this, we recently reported that systemic administration of the GCPII inhibitor 2-PMPA at a dose of 100 mg/kg daily elevated CSF NAAG levels and improved cognitive performance in EcoHIV-infected mice, accompanied by preservation of synapse and dendritic integrity [22]. However, the translational potential of 2-PMPA is limited by its highly polar chemical structure, which confers poor membrane permeability, negligible oral bioavailability, and restricted brain penetration, necessitating high systemic doses to achieve therapeutic efficacy [58].

Accumulating evidence indicates that GCPII expression is upregulated in activated microglia under neuroinflammatory conditions. Increased microglial GCPII expression has been reported in models of fetal neuroinflammation, hypoxic–ischemic encephalopathy, and cerebral palsy, where microglia-targeted GCPII inhibition modulates microglial morphology and function [26,59,60,61]. In addition, microglial- or macrophage-targeted GCPII inhibition improves cognitive performance in rodent models of multiple sclerosis and delays neuromuscular junction degeneration in amyotrophic lateral sclerosis [23,28]. These findings are particularly relevant in HIV, as microglia and brain macrophages represent the primary CNS reservoirs for HIV-1 even under suppressive cART, and HIV-infected microglia generate a pro-inflammatory microenvironment that disrupts neuronal function [18,20].

Accumulating evidence indicates that persistent microglial activation contributes to the development and progression of HIV-associated neurocognitive disorders. HIV-infected microglia serve as long-lived viral reservoirs that perpetuate chronic neuroinflammation and neurotoxicity, even in the presence of antiretroviral therapy [62,63]. In vitro microglial cultures and mouse models have shown that HIV trans-activator of transcription (Tat) and envelope glycoprotein 120 (gp120) activate microglia through canonical inflammatory pathways: Tat induces NLRP3 inflammasome activation via nuclear factor kappa-light-chain-enhancer of activated B cells (NF-κB) signaling and mitochondrial stress, whereas gp120 engages Toll-like receptor 2, collectively leading to upregulation of pro-inflammatory cytokines such as IL-1β and TNF-α and thus excitotoxic stress [64,65]. Beyond direct inflammatory signaling, activated microglia contribute to glutamate dysregulation by impairing glutamate transporter function and increasing glutamate release, resulting in excitotoxic neuronal injury [66]. Additionally, HIV-infected microglia secrete factors that promote aberrant synaptic pruning and dendritic simplification, leading to disruption of neural circuitry critical for cognitive function [67]. These indirect mechanisms, like chronic inflammation, glutamate-mediated excitotoxicity, and abnormal synaptic remodeling, collectively impair neuronal integrity without requiring direct viral infection of neurons, thereby driving cognitive decline in HIV-associated neurocognitive disorders. Consistently, positron emission tomography using translocator protein radioligands showed increased microglial activation in the frontal cortex and striatum, which correlates with cognitive deficits [68,69].

In this study, we employed a hydroxyl PAMAM dendrimer-conjugated GCPII inhibitor, D-2-PMPA, administered to EcoHIV-infected mice at a dose of 20 mg/kg (2-PMPA equivalent) every other day, which is an 8.3-fold lower dose than that used for unconjugated 2-PMPA in our prior study [22]. Consistent with prior studies [23,24,27,70], Cy5-D-2-PMPA preferentially colocalized with Iba1-positive microglia, with minimal localization to astrocytes or neurons, supporting effective microglial targeting. This observation is further supported by our previously published quantitative fluorescence-activated cell sorting (FACS) analysis of Cy5-labeled dendrimer conjugates in the mouse brain, which demonstrated that microglia exhibited an approximately 17.5-fold higher proportion of Cy5-positive cells compared to astrocytes and a 10.6-fold higher proportion compared to neurons [25]. These quantitative findings confirm that hydroxyl PAMAM dendrimers are selectively internalized by microglia in vivo, consistent with the immunofluorescent colocalization observed in the present study. Systemic administration of D-2-PMPA robustly elevated CSF NAAG levels, confirming pharmacodynamic target engagement in vivo. Importantly, D-2-PMPA treatment improved performance across multiple behavioral domains relevant to HIV-associated neurocognitive impairment, including recognition memory, social behavior, and cue-dependent fear memory, without affecting locomotor activity or anxiety-like behavior. These functional improvements were accompanied by preservation of synaptic and dendritic markers in the cortex, comparable to those observed with 2-PMPA (100 mg/kg daily), demonstrating that D-2-PMPA (20 mg/kg 2-PMPA equivalent, every other day) achieves similar neuroprotective effects at a substantially lower dose.

In this study, there are several limitations. First, while the EcoHIV mouse model recapitulates key features of HIV-associated neuroinflammation and cognitive impairment, it does not fully replicate the complexity of human HIV infection. Future studies will include evaluating the efficacy of D-2-PMPA in humanized mouse models of HIV infection, which more closely recapitulate human immune responses and viral dynamics. Additionally, Iba1 was used to identify microglial localization, however, this marker does not distinguish resident microglia from infiltrating peripheral macrophages. Future studies incorporating homeostatic microglial markers such as TMEM119 or P2RY12 would provide greater cellular specificity. Furthermore, an empty-dendrimer control group was not included in this study. Although, extensive prior work demonstrates that hydroxyl PAMAM dendrimers are biocompatible, minimally toxic, and do not independently confer cognitive or structural benefits in comparable experimental settings [23,71,72,73,74], inclusion of a dendrimer-only control in future studies will be important to definitively exclude scaffold-related effects and further strengthen causal attribution.

In summary, dendrimer-conjugated 2-PMPA demonstrated preferential uptake by microglia, robustly increased CSF NAAG concentrations, reversed cognitive and social deficits, and preserved synaptic integrity in EcoHIV-infected mice. These findings emphasize the therapeutic promise of targeting microglial GCPII for the treatment of HIV-associated neurocognitive impairment and demonstrate that hydroxyl dendrimers represent an effective delivery platform for achieving targeted CNS drug distribution.

## Data Availability

Data will be made available upon request.
